# Genetics, personality and wellbeing. A twin study of traits, facets and life satisfaction

**DOI:** 10.1038/s41598-018-29881-x

**Published:** 2018-08-17

**Authors:** Espen Røysamb, Ragnhild B. Nes, Nikolai O. Czajkowski, Olav Vassend

**Affiliations:** 1 Department of Psychology, University of Oslo, Oslo, Norway; 2 Norwegian Institute of Public Health, Oslo, Norway

## Abstract

Human wellbeing is influenced by personality traits, in particular neuroticism and extraversion. Little is known about which facets that drive these associations, and the role of genes and environments. Our aim was to identify personality facets that are important for life satisfaction, and to estimate the contribution of genetic and environmental factors in the association between personality and life satisfaction. Norwegian twins (N = 1,516, age 50–65, response rate 71%) responded to a personality instrument (NEO-PI-R) and the Satisfaction With Life Scale (SWLS). Regression analyses and biometric modeling were used to examine influences from personality traits and facets, and to estimate genetic and environmental contributions. Neuroticism and extraversion explained 24%, and personality facets accounted for 32% of the variance in life satisfaction. Four facets were particularly important; anxiety and depression in the neuroticism domain, and activity and positive emotions within extraversion. Heritability of life satisfaction was 0.31 (0.22–0.40), of which 65% was explained by personality-related genetic influences. The remaining genetic variance was unique to life satisfaction. The association between personality and life satisfaction is driven mainly by four, predominantly emotional, personality facets. Genetic factors play an important role in these associations, but influence life satisfaction also beyond the effects of personality.

## Introduction

Human wellbeing and life satisfaction are influenced by life events, health, economy and social relations^1,2^. Life satisfaction is also closely connected to personality traits^3,4^, but the nature of this relation is partly unknown. There is limited knowledge about which specific aspects, or facets, of personality are most important. Further, both personality and life satisfaction are influenced by genetic factors^5–8^, but we have inadequate understanding of the role of genetic and environmental factors in explaining the links between personality and life satisfaction. Which traits, and which particular facets, are most important for promoting or obstructing individual life satisfaction? Are the associations accounted for by genetic factors, environmental factors, or both? Does the genetic influence on life satisfaction stem entirely from personality related genetics, or do genetic factors for life satisfaction operate independently of personality?

The scientific study of the good life and wellbeing has prospered in recent years^9–11^. As the field has grown, a number of constructs and approaches have emerged. The construct of subjective wellbeing (SWB) occupies a central position, and is typically seen as comprising three components - frequent positive affect, infrequent negative affect, and presence of life satisfaction^12^. Life satisfaction represents a global evaluation of life, a mental summarizing of life as good, or not so good, according to the individual’s own values, norms, and ideals^13^. As such, life satisfaction constitutes the key cognitive component in SWB and positive mental health^12,14^. In parallel with SWB, the construct of psychological wellbeing (PWB) contains components such as engagement, personal growth, and flow-experiences, thereby focusing more on functioning well than feeling well^15–17^. Research on SWB and PWB represent two different, but complimentary traditions, focusing on distinguishable yet related dimensions of wellbeing overall. The dimensions of SWB and PWB have also been integrated into broader models, such as the tripartite model of mental wellbeing (MWB) including emotional, psychological and also social wellbeing^18^. Thus, in correspondence with research on taxonomies and the nature of psychopathology (i.e., illbeing), the wellbeing field today addresses several aspects of the good life. Life satisfaction represents a central component in SWB in particular, but features as an important aspect inherent in most models.

## Genetics of Wellbeing

Genetic factors appear to play an important role in most human characteristics^19^ and wellbeing is no exception. Heritability estimates for different conceptualizations of wellbeing typically range from 0.30 to 0.50^20–25^. A meta-analysis of 13 studies from seven different countries and including more than 30,000 twins, reported a weighted average heritability of 0.40 for wellbeing^5^. This meta-analysis also found substantial heterogeneity in heritability estimates across studies, beyond that expected by random fluctuations, thus verifying the theoretical notion that there is no fixed heritability for wellbeing. Rather, the share of variance accounted for by genetic factors varies across cultures, age groups, and the particular wellbeing phenomena studied. Another meta-analysis by Bartels^6^, with somewhat different inclusion criteria, samples and analytic strategy reported an average heritability of 0.36 for wellbeing.

There is evidence of a common genetic influence on different wellbeing components such as subjective happiness, life satisfaction, SWB and PWB^24,26^, but also genetic influences that are specific to the different components^26,27^. The genetic factors in wellbeing are partly related to the genetic influences on social support^28^, and inversely, depression^29^ and internalizing disorders^30–32^. Additionally, longitudinal studies have shown that genetic factors account for most of the stability in wellbeing with heritability for the stable variance, or dispositional wellbeing, estimated in the 70–90% range^33,34^. By contrast, environmental factors constitute the major source of change in wellbeing^34,35^.

Despite clear evidence of substantial genetic influences on wellbeing in general, findings on life satisfaction are somewhat divergent, with heritability estimates ranging from zero to 0.59^24,32,35–38^. The meta-analysis by Bartels^6^ examined heritability of life satisfaction specifically, and reported an average heritability of 0.32. Thus, life satisfaction appears to be somewhat more influenced by environmental factors than other dimensions of wellbeing. Further, although levels of life satisfaction commonly vary only moderately with age, there might be age-related moderation of genetic and environmental factors. As life satisfaction represents an evaluation of life-so-far, life at older age likely include more life events, adversaries and accomplishments than life at younger age, thereby suggesting stronger environmental than genetic effects. There is a need for more knowledge about the genetic and environmental influences on life satisfaction in the mature population, measured by validated and reliable multi-item instruments.

Recent advances in molecular genetics have contributed to our understanding of the genetic underpinnings of wellbeing – including heritability. Genome-wide Complex Trait Analysis (GCTA) uses genotyping of common Single Nucleotide Polymorphisms (SNPs) in unrelated individuals to estimate heritability. Rietveld, *et al*.^39^ reported that up to 18% of the variance in wellbeing can be explained by cumulative additive effects of genetic variants that are frequent in the population. This suggests that common genetic polymorphisms account for nearly half of the overall heritability of SWB. Genome-Wide Association Studies (GWAS) are used to identify specific genetic variants associated with a phenotype. Recently, Okbay, *et al*.^40^ used GWAS in a sample of 298,420 individuals, and identified three credible genetic loci associated with wellbeing. However, these three variants explained only a small fraction (4%) of the variance. Molecular genetic studies expand rapidly and are expected to provide important new insights into the genetics of wellbeing. However, it also seems clear that twin and family studies are unique in their ability to capture the total genetic and environmental factors involved, along with the overall overlap and specificity across different characteristics.

### Personality and Life-Satisfaction

Personality refers to relatively stable and characteristic patterns of cognition, emotion, and behavior that vary across individuals. These patterns are commonly described in terms of specific personality traits. The most widely known trait models today are the five-factor and big five models^41,42^, which converge on five broad personality traits, including extraversion, neuroticism, openness, conscientiousness, and agreeableness. There is a well-established relationship between personality traits and wellbeing in general, and personality and life satisfaction in particular^3,4,43^. More specifically, the big five traits of neuroticism and extraversion consistently explain substantial amounts of variance in wellbeing. The findings are more mixed regarding the trait of conscientiousness, whereas agreeableness and openness seem to play a limited, or negligible role in wellbeing^3,43,44^.

The five-factor model of personality is hierarchical with the higher-order domains (traits) comprising a set of lower-order facets^45^. For example, in the NEO-PI perspective, as developed by Costa and McCrae^41^, the domain of neuroticism includes the facets of anxiety, hostility, depression, self-consciousness, impulsiveness and vulnerability to stress. Correspondingly, the domain of extraversion includes warmth, gregariousness, assertiveness, activity, excitement-seeking and positive emotions. Despite solid evidence for relations between the general big five factors and wellbeing, there is still limited knowledge about which facets of the traits that contribute the most to wellbeing.

Theoretically, *inter-personal facets* such as warmth and gregariousness (sociability) contribute to wellbeing indirectly by creating well-functioning social relationships that subsequently influence wellbeing. Social support and good social relations have quite consistently been found to correlate positively with wellbeing^28,46,47^, and may partly be influenced by personality traits and facets.

There is also theoretical reason to expect factors contributing to *accomplishments* and *goal attainment*, in the conscientiousness domain, to be important for life satisfaction^48–51^. Life satisfaction judgments consider the gap between actual states and ideal states. Personality facets such as competence, self-discipline, achievement-striving and dutifulness may be important in obtaining ideal states, and are thus likely to predict life satisfaction.

Finally, personality tendencies to certain *emotional* experiences, such as anxiety or positive emotions may similarly influence wellbeing as life satisfaction judgments are coloured by both current emotional states and by memories of past emotional episodes. For example, a personality disposition to experience positive emotions may contribute to many episodes of joy and enthusiasm. These episodes may constitute a basis for the subsequent evaluation of life so far^4,52,53^. Thus, from a theoretical perspective both interpersonal facets, accomplishment-related facets and emotional facets would be important in generating a good life.

Empirical examinations of relations between personality facets and life satisfaction are limited. However, a few studies have shed light on the issue. Schimmack and colleagues^52^ found the depression facet of neuroticism, and the positive emotions facet of extraversion to be the strongest and most consistent predictors of life satisfaction. They concluded that depression is more important than anxiety or anger, and a cheerful temperament is more important than being active or sociable. Quevedo and Abella^54^ found depression and the achievement striving facet of conscientiousness, but not positive emotions, to be the important facets, whereas Albuquerque, *et al*.^55^ identified depression and positive emotions as central, and found an additional effect from the vulnerability facet of neuroticism. Finally, Anglim and Grant^56^ reported significant semi-partial correlations between life satisfaction and the three facets of depression, self-consciousness and cheerfulness.

These studies have provided important knowledge about the nuanced associations between personality and life satisfaction, and point to some particularly important personality facets. Yet, findings so far are limited, as the results are partly divergent, and mostly based on (young) student samples and convenience samples. Consequently, there is a need for replication of findings and expansion of cultures and age groups studied.

### Genetic and Environmental Factors in Personality and Life Satisfaction

Personality traits are relatively stable characteristics, and there is considerable evidence for genetic components^57,58^. Although associations between personality traits, and partly their facets, and wellbeing are established, there is limited knowledge about the mechanisms involved in these associations. Are the associations between personality and wellbeing due to common genetic factors, and is the entire heritability of wellbeing accounted for by the genetic factors in personality – is wellbeing genetically speaking a personality thing?

A few studies have addressed these questions at the level of broad personality traits. First, Weiss and colleagues^59^ found a global SWB-measure to be accounted for by unique genetic effects for neuroticism, extraversion and conscientiousness, and by a common genetic factor that influenced all five personality domains. Environmental factors also contributed to the associations, but there were no genetic effects unique to SWB. In a similar vein, Hahn and colleagues^38^ reported shared genetic effects for life satisfaction and the traits of neuroticism and extraversion, but not conscientiousness. Both additive and non-additive genetic effects contributed to the relation between personality and life satisfaction, and again the entire heritability of life satisfaction was accounted for by personality-related genetic factors. Finally, a study examining personality traits and flourishing found substantial genetic effects on the associations, but also identified a unique genetic influence on wellbeing, unrelated to personality^60^. This latter study was unique in its focus on the construct of flourishing as comprising both eudaimonic and hedonic aspects of wellbeing, based on Keyes’ tripartite model including emotional, psychological and social wellbeing^61^, and thereby also involving both feeling good and functioning well.

Thus, a few recent studies have reported exciting evidence of a substantial genetic contribution to the association between personality traits and wellbeing. However, several important questions remain to be addressed. First, no studies to date have examined genetic and environmental contributions to the associations between personality facets and wellbeing. Given the findings for broad personality traits, we hypothesize considerable genetic effects also for their facets. Yet, the magnitude of such effects is unknown. Second, only one study^38^ has examined life satisfaction specifically – rather than global measures of wellbeing. Third, as previous studies have relied only on short-form measures of broader traits, there is a pressing need for examining both traits and facets in relation to life satisfaction by means of comprehensive, valid, well-established instruments. Fourth, findings from the few previous studies are divergent as to whether the entire genetic effect on wellbeing is due to personality-related genetic influences. Fifth, whereas prior studies have examined samples with broad age ranges, we wanted to examine a specific period in life – middle to late adulthood – to assess how relatively stable personality characteristics contribute to life satisfaction in a life course perspective. Finally, as previous studies have been inconclusive regarding sex-differences in the underlying etiology of wellbeing^6,62^, we also wanted to test for such differences.

The aims of the current study were to (a) identify personality traits and facets that contribute uniquely to life satisfaction, and thereby pinpoint the dispositional constituents of a happy personality, (b) estimate the heritability of life satisfaction in middle to late adulthood, (c) disentangle the genetic and environmental influences shared by personality traits/facets, and life satisfaction, and finally hence to (d) determine whether all of the genetic influence on life satisfaction is due to personality related genetic factors as suggested by some previous studies.

## Results

### Correlation and Regression Analyses

As shown in Table 1, neuroticism, extraversion, and conscientiousness were all significantly correlated with life satisfaction, while agreeableness and openness were not. The strongest correlation was found for neuroticism, yet with substantial associations also for extraversion and conscientiousness. In the multiple regression analysis including these three factors, only neuroticism and extraversion showed significant unique contributions. The effects remained when controlling for sex and age. A total of 24% of the variance in life satisfaction was accounted for.

**Table 1:** Descriptives and associations between big five traits and life satisfaction.

	Descriptives			Associations	
	Range	Mean	sd	Corr	*β*
Neuroticism	1–5	1.58	0.42	−0.48**	−0.41**
Extraversion	1–5	2.22	0.35	0.30**	0.10**
Openness	1–5	2.12	0.35	0.02	
Agreeableness	1–5	2.70	0.27	0.05	
Conscientiousness	1–5	2.56	0.31	0.28**	0.05
*R* ^2^					0.24**
Life satisfaction	1–7	5.19	1.22		

We next examined the associations for all the 30 personality facets. Table 2 shows the resulting correlations. A total of 23 facets were significantly associated with life satisfaction. In the neuroticism domain, all facets showed significant correlations, ranging from −0.14 for impulsiveness to −0.51 for depression. Within extraversion, excitement seeking was virtually unrelated (0.05) to life satisfaction, whereas positive emotions (0.30) and activity (0.28) showed substantial associations. In the openness domain, only one facet, ideas, showed a significant but very modest correlation (0.08). The agreeableness domain was notable for a combination of positive and negative associations. Trust (0.17) and altruism (0.09) were positively associated with life satisfaction, while negative associations were shown for modesty (−0.08) and tendermindedness (−0.08). Finally, in the conscientiousness domain, all factors showed significant and positive correlations, and in particular competence (0.30) and self-discipline (0.28) appeared to be potentially important.

**Table 2:** Descriptives and associations between personality facets and life satisfaction.

	Descriptives		Associations			
	Mean	sd	Corr		*β*	
**Neuroticism**						
N1 Anxiety	1.56	0.67	−0.44	**	−0.**15**	**
N2 Hostility	1.39	0.51	−0.31	**		
N3 Depression	1.66	0.64	−0.51	**	−0.**35**	**
N4 Self-Consciousness	1.72	0.53	−0.29	**		
N5 Impulsiveness	1.84	0.48	−0.14	**	0.06	*
N6 Vulnerability to stress	1.30	0.46	−0.40	**		
**Extraversion**						
E1 Warmth	2.74	0.43	0.17	**		
E2 Gregariousness	2.36	0.56	0.13	**		
E3 Assertiveness	1.87	0.57	0.25	**		
E4 Activity	2.36	0.51	0.28	**	0.**12**	**
E5 Excitement seeking	1.51	0.51	0.05			
E6 Positive emotion	2.50	0.56	0.30	**	0.**16**	**
**Openness**						
O1 Fantasy	1.76	0.51	−0.05			
O2 Aesthetics	2.21	0.65	−0.01			
O3 Feelings	2.37	0.46	−0.01			
O4 Actions	1.89	0.48	0.06		−0.07	*
O5 Ideas	2.02	0.60	0.08	**		
O6 Values	2.47	0.41	0.03		−0.09	**
**Agreeableness**						
A1 Trust	2.81	0.40	0.17	**		
A2 Straightforwardness	2.81	0.44	0.02			
A3 Altruism	2.95	0.38	0.09	**		
A4 Compliance	2.45	0.45	0.07	*	0.07	*
A5 Modesty	2.66	0.44	−0.08	**		
A6 Tendermindedness	2.56	0.36	−0.08	**		
**Conscientiousness**						
C1 Competence	2.71	0.38	0.30	**		
C2 Order	2.33	0.44	0.09	**	−0.07	*
C3 Dutifulness	3.05	0.39	0.13	**		
C4 Achievement striving	2.38	0.47	0.24	**		
C5 Self-Discipline	2.56	0.47	0.28	**		
C6 Deliberation	2.30	0.47	0.15	**	0.09	**

Note: N1, N2, etc refer to Neuroticism facet number 1, 2, etc.

*p < 0.05; **p < 0.01; Adjusted *R*^2^ = 0.32.

Next, regression analyses were conducted in which all 30 facets were tested simultaneously. Ten facets showed significant and unique effects (Table 2). In total, these facets explained 33% of the variance (adjusted R^2^ = 32%) in life satisfaction. Four facets yielded substantial betas, that is above 0.10, and with p < 0.01, namely N1-anxiety, N3-depression, E4-activity and E6-positive emotions (label N1 refers to Neuroticism facet 1, etc). The remaining significant facets were found across all personality domains and included the openness facets of values and actions, the agreeableness facet of compliance, and the conscientiousness facets of order and deliberation. However, these effects were relatively minor (i.e., beta <0.10). Also, when performing Bonferroni correction and examining the False Discovery Rate^63^, only the four facets with betas >0.10 retained p < 0.01. Thus, for the biometric analyses disentangling genetic and environmental effects we focused on these four facets with substantial and significant effects. Summarizing the regression findings, the happy, or satisfied personality is given by the equation:

$$\begin{array}{rcl}Happy\,personality & = & 0.12\,\ast \,Activity+0.16\,\ast \,Positive\,emotions\\  &  & -0.15\,\ast \,Anxiety-0.35\,\ast \,Depression\end{array}$$

### Biometric twin analyses

Twin-cotwin correlations across zygosity groups were calculated for the neuroticism and extraversion traits, the four major facets (i.e., anxiety, depression, positive emotion, activity) and life satisfaction. Table 3 shows the correlations. In general, the monozygotic (MZ) correlations were substantial, and in all cases higher than the corresponding dizygotic (DZ) correlations, indicating additive genetic effects.

**Table 3:** Twin-cotwin correlations for life satisfaction, personality traits, and facets.

	MZ	DZ	MZM	DZM	MZF	DZF
Life satisfaction	0.31**	0.15**	0.35**	0.05	0.29**	0.17**
**Big five traits**						
Neuroticism	0.56**	0.27**	0.52**	0.12	0.55**	0.27**
Extraversion	0.46**	0.27**	0.42**	0.15	0.49**	0.32**
**Facets**						
N1-Anxiety	0.52**	0.24**	0.46**	0.00	0.52**	0.25**
N3-Depression	0.48**	0.24**	0.47**	0.16*	0.47**	0.24**
E4-Activity	0.39**	0.26**	0.38**	0.11	0.40**	0.32**
E6-Positive emotions	0.40**	0.19**	0.42**	0.21**	0.37**	0.18**

MZ = Monozygotic; DZ = Dizyogotic; M/F = Males/Females, *p < 0.05; **p < 0.01.

**Figure 1:**
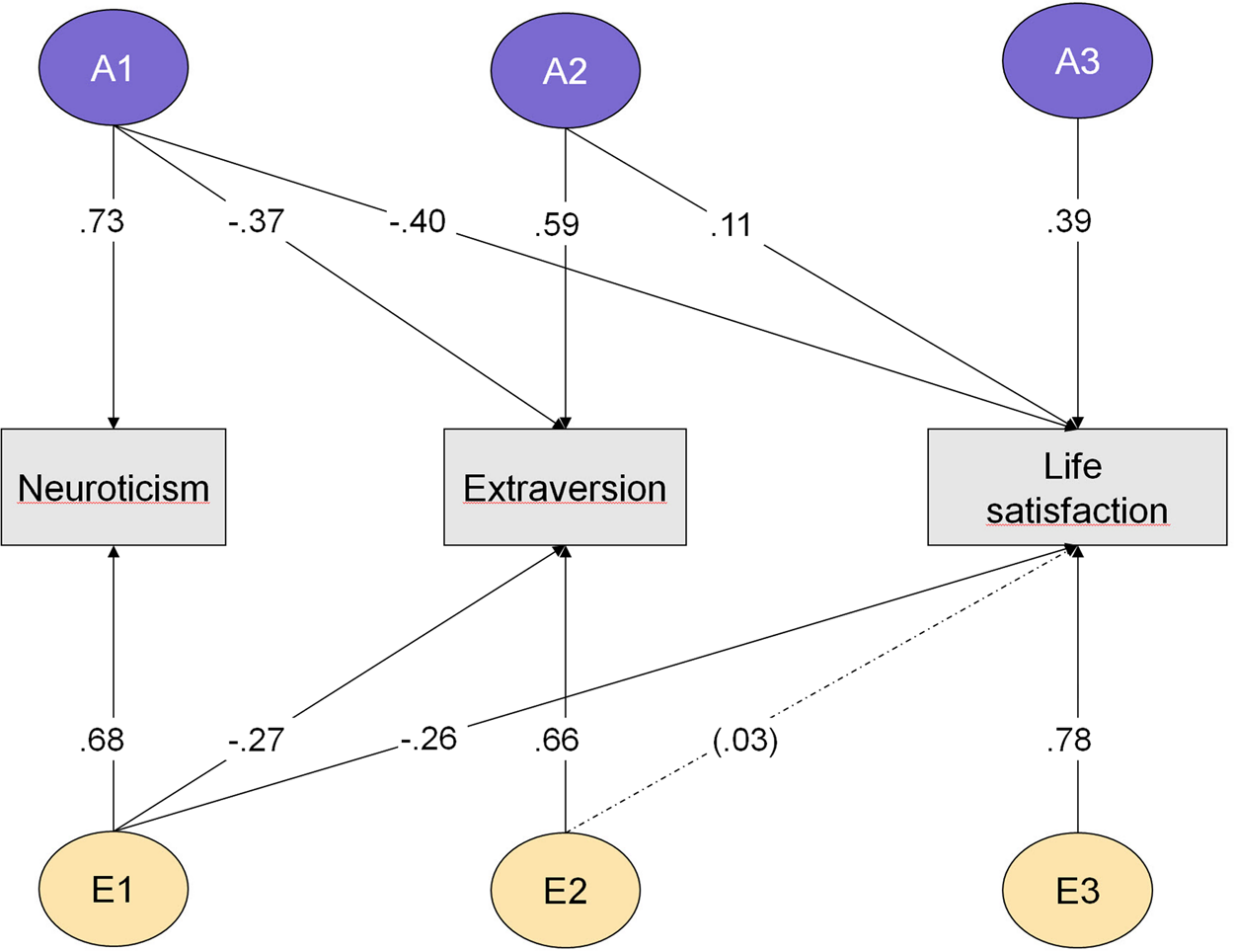
Biometric Cholesky model of neuroticism, extraversion and life satisfaction. A = Additive genetic factor; E = Non-shared environmental factor; All parameters: p < 0.05, except one parameter (n.s.) in parenthesis and dotted arrow.

Based on the findings from the regression analyses, we next tested a set of tri-variate Cholesky models including neuroticism, extraversion and life satisfaction. Table 4 (upper part, block I) shows the fit of the different models. Model 1 included additive genetic (A), common environmental (C) and non-shared environmental (E) factors, and allowed estimates to vary across sex. Model 2, which included only A and E effects, did not fit significantly worse (i.e., Δ − 2LL = 0.89, Δdf = 12, n.s.) and produced a lower AIC value. Further, models 3 and 4, involving scalar sex-limitation, yielded additional improvements in fit, that is, increasingly lower AIC values, no significant reduction in fit, and more parsimony. Finally, models 5 and 6, where parameters were constrained to be equal across sex, resulted in higher AIC and worse fit. Thus, model 4 yielded overall best fit, and included only A and E effects with standardized parameters similar for men and women. Figure 1 shows the Cholesky parameters of the model.

**Table 4:** Model fit for multivariate Cholesky models of personality and life satisfaction.

	−2LL	df	Δ − 2LL	Δdf	p	AIC
**I. Big five factors**						
1. Common sex-lim ACE	6570.32	4493	—	—	—	−2415.68
2. Common sex-lim AE	6571.21	4505	0.89	12	0.99	−2438.79
3. Scalar sex-lim ACE	6583.22	4508	7.98	15	0.61	−2432.78
**4**. **Scalar sex-lim AE**	**6583**.**55**	**4514**	**8**.**31**	**21**	0.**90**	−**2444**.**45**
5. Equal across sex ACE	6593.55	4511	18.31	18	0.18	−2428.45
6. Equal across sex AE	6593.87	4517	18.63	24	0.49	−2440.13
**II. Personality facets**						
7. Common sex-lim ACE	13046.14	7460	—	—	—	—1873.86
8. Common sex-lim AE	13058.45	7490	12.27	30	0.99	−1921.55
9. Scalar sex-lim ACE	13082.11	7500	36.51	40	0.70	−1917.30
**10**. **Scalar sex-lim AE**	**13084**.**62**	**7515**	**38**.**48**	**55**	0.**96**	−**1945**.**38**
11. Equal across sex ACE	13108.86	7505	62.68	45	0.04	−1901.14
12. Equal across sex AE	13110.01	7520	63.86	60	0.34	−1930.00

*Note*. Block I: Tri-variate model with neuroticism, extraversion and life satisfaction

Block II: Five-variate model with anxiety, depression, activity, positive emotions and life satisfaction.

Common sex-limitation: sex specific parameters.

Scalar sex-limitation: standardized parameters equal across sex.

Equal across sex: No sex-limitation, parameters constrained to be equal across sex.

−2LL = minus 2 LogLikelihood; p-values from test of difference from base models (1 and 7).

Best-fitting models indicated in bold.

Heritability estimates (with 95% CIs) were 0.53 (0.46; 0.60) for neuroticism, 0.49 (0.41; 0.56) for extraversion and 0.32 (0.23; 0.41) for life satisfaction. Based on the Cholesky model we also calculated the genetic and environmental correlations between the two personality traits and life satisfaction. Genetic correlations were −0.70 (−0.58; −0.83) for neuroticism, and 0.53 (0.37; 0.68) for extraversion. Correspondingly, environmental correlations were −0.32 (−0.23; −0.40) for neuroticism and 0.15 (0.08; 0.24) for extraversion.

**Figure 2:**
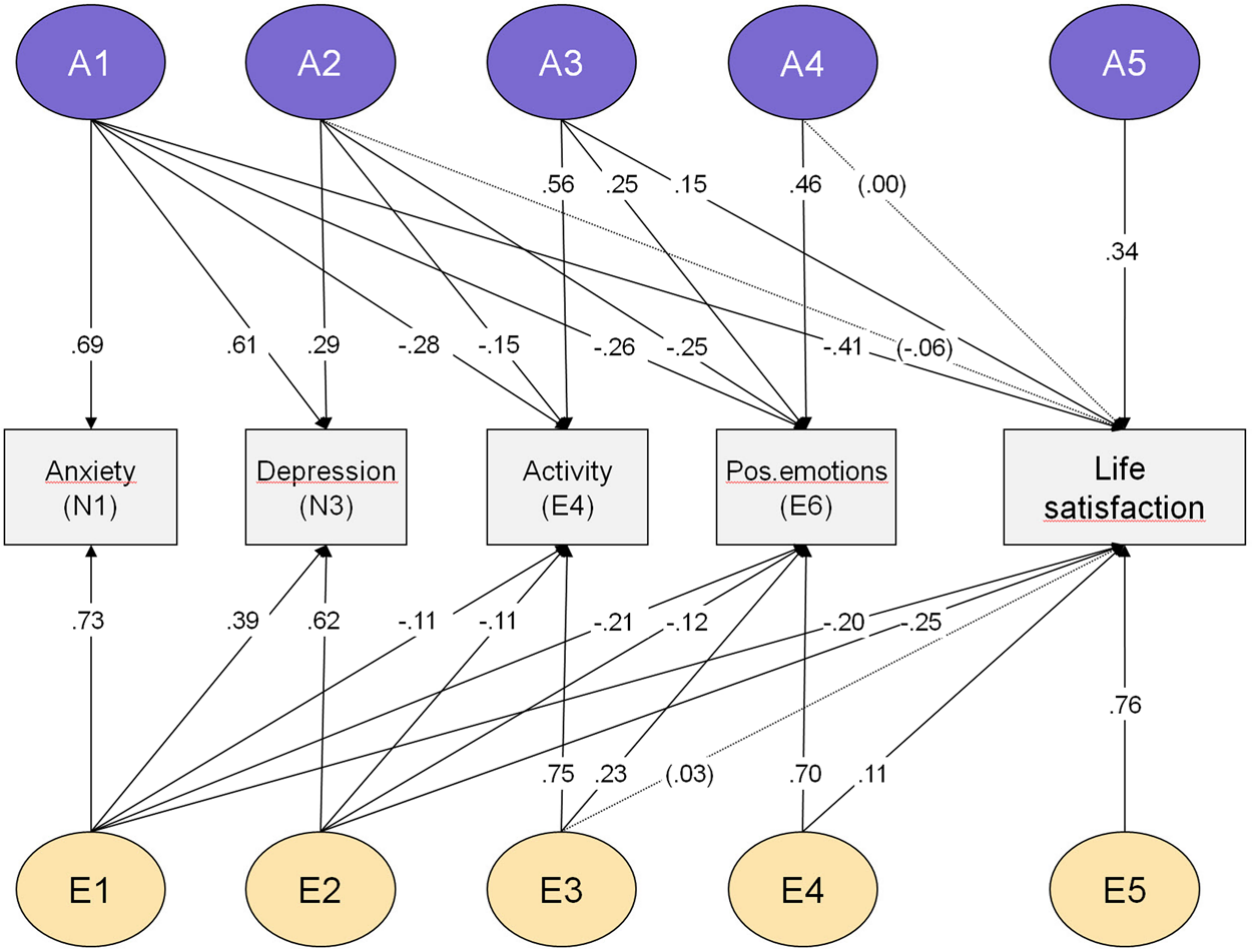
Biometric Cholesky model of four personality facets (anxiety, depression, activity and positive emotions) and life satisfaction. A = Additive genetic factor; E = Non-shared environmental factor; N = Neuroticism; E = Extraversion. All parameters: p < 0.05, except three parameters in parentheses and dotted arrows.

Moving from the big five factors to the personality facets, again we tested a set of models including the four facets found to be most strongly predictive of life satisfaction. Table 4 (lower part, block II, models 7–12) shows the results. Again, the best fitting model included only A and E effects (model 10), and standardized estimates did not differ across sex. Figure 2 shows the parameter estimates of the best model.

In this best-fitting model, heritabilities were estimated to 0.47 (0.40–0.54) for anxiety, 0.46 (0.38–0.53) for depression, 0.42 (0.33–0.49) for activity, 0.40 (0.32–0.48) for positive emotions, and 0.31 (0.22–0.40) for life satisfaction. As can be seen in Fig. 2, genetic factors from both the neuroticism and extraversion facets uniquely influenced life satisfaction. However, after the effect of latent factor A1 (reflecting the genetic variance in anxiety) was accounted for, there was no additional genetic effect from the unique genetic factor of depression (A2). Likewise, the genetic variance in activity (A3) influenced life satisfaction, but there was no additional genetic effect from positive emotions (E4). Thus, the genetic variance in each of the two personality domains, which influenced life satisfaction, appeared to be shared by the facets within their respective domain (neuroticism or extraversion), and the facet-specific influences on life satisfaction appeared to be driven by environmental effects. Notable is also the unique genetic factor (A5) influencing life satisfaction after all the genetic effects of the facets were accounted for.

**Figure 3:**
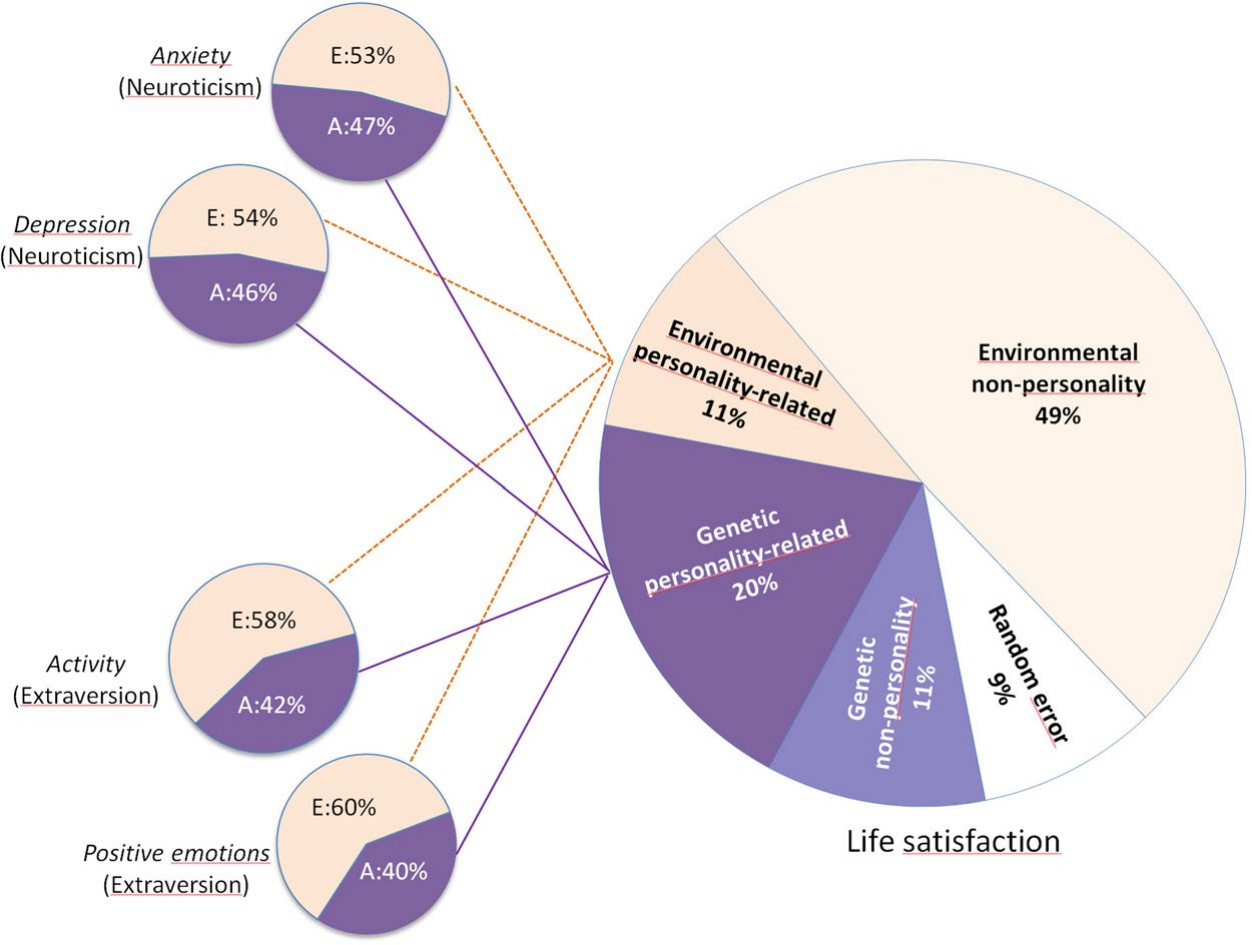
Life satisfaction: Sources of origin decomposed. Genetic and non-shared environmental components, divided into personality-based and non-personality sources. Estimated random error (1-α) also shown for life satisfaction. For facets, additive genetic (A) and non-shared environmental variance (E) shown.

A total of 20% of the variance in life satisfaction was accounted for by personality-related genetic factors, and 11% was explained by a genetic factor unrelated to personality. Thus, of the total heritability of life satisfaction (*h*^2^ = 0.31), about 65% was driven by personality genetic factors, and the remaining 35% was due genetic influences independent of personality. Further, the combined effect of personality facets on life satisfaction also involved environmental effects, accounting for 11% of the variance. Finally, 58% of the variance in life satisfaction was environmental in origin and unrelated to personality. This environmental component includes random measurement error (1-alpha = 9%), thus implying an estimated true non-shared environmental component of 49%. Figure 3 shows the decomposed sources of variance for life satisfaction, along the corresponding variance components of the four facets.

**Figure 4:**
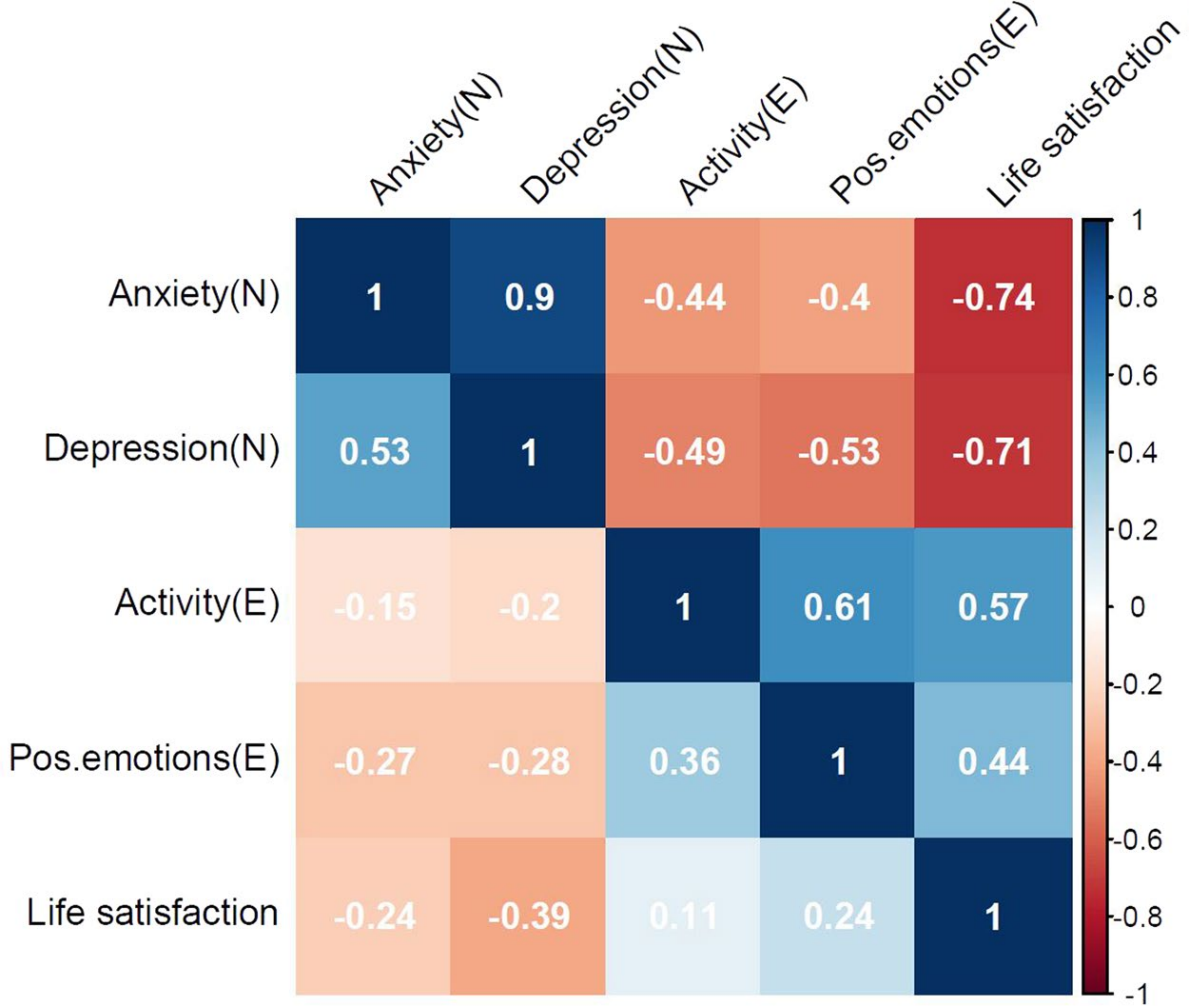
Genetic and environmental correlations, above and below diagonal, respectively.

Based on the best-fitting model, we also calculated genetic and environmental correlations for the variables, shown in Fig. 4, above and below the diagonal, respectively. Generally, the genetic correlations within personality domains were high, and the genetic correlations between facets and life satisfaction were moderate to high. The corresponding environmental correlations were generally lower, but suggested also important associations due to environmental factors.

## Discussion

We set out to delineate etiological factors involved in the associations between personality and life satisfaction. Personality traits are well-established predictors of wellbeing in general and life satisfaction in particular^3,43^. The issue of why personality traits influence life satisfaction was addressed along two paths: First, we examined the broad personality traits and the specific personality facets that drive the effects from traits. Second, we examined the role of genetic and environmental factors in the link between personality and life satisfaction.

### Personality and life satisfaction

At the level of broad traits, neuroticism and extraversion were uniquely predictive of life satisfaction, in line with previous studies^3,4^. Further, four facets of unique importance for life satisfaction were identified, namely anxiety and depression from the neuroticism domain, and positive emotions and activity from the extraversion domain. The happy, or satisfied personality thus seems to have low levels of anxiety and depression, and high levels of positive emotions and activity. The highly emotional nature of these facets is noteworthy. That is, three out of the four facets explicitly refer to affective tendencies, whereas the fourth facet (activity) adds vigor, energy and liveliness^41^. Thus, the cognitive evaluation of life satisfaction is partly based on emotional tendencies inherent in the big five model. Our findings accord with previous studies in identifying depression, and partly positive emotions, as central predictors of life satisfaction^52,55^. However, whereas prior studies have found facets such as vulnerability, excitement-seeking^52^, and achievement striving^54^ to be significant, in this population based sample covering middle to late adulthood, we found anxiety and activity to be important.

Although a high number of facets were correlated with life satisfaction at the zero-order level, most facets did not show unique effects on life satisfaction in the multivariate analyses. There were no unique effects from interpersonal facets such as warmth, assertiveness, gregariousness, trust or straightforwardness. Neither did we find effects from accomplishment-related facets such as competence, self-discipline or dutifulness. This does not imply that having warm and trustful relations, or high levels of competence, are inconsequential for wellbeing. Rather, we interpret the findings to suggest that the predominantly emotional facets are underlying tendencies accounting for some of the zero-order associations between other facets and life satisfaction.

Why and how do depression, anxiety, positive emotions and activity play such important roles in generating a good – or not so good – life? We believe that a dual set of mechanisms are involved. First, from a top-down perspective^64,65^, life satisfaction is influenced by a general way of seeing life, the glasses through which we perceive the world. Therefore, negative and positive affective tendencies might color our ongoing evaluations of what life has been like.

Second, and in accordance with a bottom-up perspective^64^, positive and negative affective tendencies over time contribute to life experiences that are taken into account when performing a current evaluation. That is, a person with a strong tendency to experience positive emotions and activity/energy, combined with a low tendency to depression and anxiety, might recall a high number of episodes characterized by such experiences, and thereby summarize life as mostly good. In contrast, a person prone to anxiety and depression, who experiences few positive emotions and low activity/energy, might have a mental album comprising of numerous episodes and life periods that are less satisfactory.

Importantly, depression (sadness/distress), anxiety (fear) and positive emotions are represented in most models of basic emotions^66–69^. These basic emotions are seen as evolutionary adaptive and functional responses to environmental exposures. Although we are all equipped with the potential to experience such emotions, from a personality perspective there are individual differences in our tendency to activate them, and as such they are encompassed as facets in the five-factor personality model. Adding the facet of activity (energy) to the equation we have four basic building blocks, inherent in our personality, that contribute uniquely to a good life. In a dual-process model these tendencies operate both by coloring current perceptions of life-so-far, and by having contributed to a number of positive and/or negative experiences throughout the life lived.

In the wellbeing-illbeing structural model (WISM), wellbeing is conceptualized as comprising both well-staying and well-moving, and illbeing is correspondingly divided into ill-staying and ill-moving^15,23^. The model posits that humans have various goal states, and we may experience the presence of an obtained goal state (well-staying), we may be in a process towards a desired goal (well-moving), we may experience threats implying a risk of losing goals (ill-moving), and finally we may realize that a goal state is lost (ill-staying). The current findings are noteworthy in identifying personality facets that have certain connections to these four goal-state conditions. Positive emotions can be seen as indicative of well-staying, activity is potentially important for well-moving, anxiety is a core feature of ill-moving and depression is a characteristic of loss and ill-staying. Thus, our findings lend support to the notion of well-staying, well-moving, ill-staying and ill-moving as fundamental human scenarios that all are important for generating or obstructing good lives.

### The role of genetic and environmental factors

The estimated heritability for life satisfaction was 0.31. This is in the lower range of previous estimates for general wellbeing^24,35^, and below a meta-analysis estimate of 0.40^5^. However, although findings are divergent, several studies have reported heritability estimates for life satisfaction that are moderately lower than for other wellbeing constructs^32,70,71^, and the meta-analysis by Bartels^6^ reported a heritability of 0.32 for life satisfaction. Our study is one of the first to examine life satisfaction beyond midlife specifically, with a well-established instrument. The findings point to both genetic and environmental influences – yet with the latter clearly being the most important. As such, life satisfaction appears to be more about the environmentally influenced life course, events and relationships, than about a genetically driven tendency. Such an interpretation also implies potentials for change in life satisfaction, and possibly substantial benefits of wellbeing interventions^35,72^.

We tested models examining sex-differences in the genetic and environmental sources of wellbeing. In line with several studies^6,21,70^, but in contrast to some others^62,73^, we found the heritability, and the environmental component, to be of similar magnitude for females and males. Although the total variance might vary, our findings provide evidence that the relative contribution of genetic factors is similar across sex.

While genetic factors seem to play only a moderate role for the total variability in life satisfaction, genetic factors appear to have a major role in the association between personality and life satisfaction. Both at the levels of broad traits and more specific facets, genetic factors were highly important in explaining the effect of personality on life satisfaction. That is, there are genetic factors influencing personality that also influence life satisfaction, whereas environmental factors play a more limited role in this relationship. More specifically, the genetic dispositions to experience a low degree of depression and anxiety, and a high degree of positive emotions and activity contribute to a life experienced as good and satisfactory.

To our knowledge this study is the very first to examine genetic factors in the association between personality facets and life satisfaction. In general, our finding of genetic factors playing a key role accord with the few previous studies examining broad personality traits and wellbeing in genetically informative samples^38,59,74^. However, whereas two of these previous studies found the entire heritability of wellbeing to be due to personality-related genetic factors^38,59^, in line with Keyes *et al*.^60^ we identified a unique genetic factor influencing life satisfaction beyond the effect of personality, accounting for 11% of the total variance. We can only speculate on the genetic mechanisms involved. Theoretically, there could be a specific, genetically driven, tendency to having a positive outlook on life that is not captured within the five-factor model. Alternatively, there could be influences from conditions such as mental abilities or somatic disorders – both of which have substantial genetic influences^19^ – that also are outside the personality domain. Further studies are required both to address this aspect of life satisfaction, and generally to delineate the complex processes starting with DNA-molecules and ending up with a person evaluating her life as good – or not.

The findings also accord with a recent molecular genetic study of the association between wellbeing and neuroticism. Okbay, *et al*.^40^ used GWAS and bivariate Linkage Disequilibrium Score regression, and reported a genetic correlation of −0.75 between wellbeing and neuroticism. Despite the limited variance explained in the GWAS it is noteworthy that the correlation corresponds highly with the current estimate of genetic correlations of −0.70 for neuroticism, −0.74 for the anxiety facet, and −0.71 for the depression facet.

It is also noteworthy that there was a common genetic factor for anxiety and depression that contributed to life satisfaction, and there was no unique genetic variance in depression that predicted life satisfaction beyond that shared with anxiety. The facet-specific influences appear to be driven by environmental effects. Corresponding findings were seen for extraversion; a common genetic factor for activity and positive emotions contributed to the genetic variance in life satisfaction.

Strengths of the current study include a population based sample, a fairly high response rate, and well-established extensive measurements. Nevertheless, some limitations should be noted. First, as with any twin study, heritabilities and genetic correlations are not fixed figures, but are estimated for a certain population, and only future studies can validate the findings across other societies and age groups. Second, the sample size implies limited ability to identify small effects – potentially common environmental factors or sex differences. Third, although the NEO-PI-R is a well-established instrument, the reliabilities of the facets were partly limited. Measurement error is captured in the E-factor in the biometric analyses, and might contribute to reduced environmental, but not genetic, correlations.

## Conclusion

The findings replicate previous studies of wellbeing and life satisfaction as influenced by genetic factors – with heritabilities in the 30–40% range^5,6^. We also replicate substantial associations between wellbeing and personality, both for the general traits of neuroticism and extraversion, and for specific facets^3,52,56^. Moreover, we identified four personality facets that appear to play an important role in driving the associations between personality and life satisfaction. These facets include basic emotional tendencies, and point to the importance of emotions as sources of direct and indirect pathways that contribute to good lives. Roughly two thirds of the genetic variance in life satisfaction was found to be due to these facets. In addition, we found a certain genetic component in life satisfaction unrelated to personality traits or facets. Finally, the findings provide solid evidence of the role of environmental factors in generating good lives – also by contributing to associations between personality and life satisfaction.

## Methods

### Sample

Twins were recruited from the Norwegian Twin Registry (NTR). The registry comprises several cohorts of twins^75,76^, and the current study drew a random sample from the cohort born 1945–1960. In 2010, questionnaires were sent to a total of 2,136 twins. After reminders, 1,516 twins responded, yielding a response rate of 71%. Of the participants, 1,272 individuals were pair responders, and 244 were single responders. Zygosity has previously been determined based on questionnaire items shown to classify correctly 97–98% of the twins^77^. The cohort, as registered in the NTR, consists only of same-sex twins, and the study sample consisted of 290 monozygotic (MZ) male twins, 247 dizygotic (DZ) male twins, 456 MZ female twins and 523 DZ female twins. The age range of the sample was 50–65 years (mean = 57.11, *sd* = 4.5). The study was approved by the Regional Committee for Medical and Health Research Ethics of South-East Norway, and informed consent was obtained from all participants. All methods were performed in accordance with relevant guidelines and regulations.

### Measures

*Life satisfaction* was measured with the Satisfaction With Life Scale (SWLS) developed by Ed Diener and colleagues^78,79^. The SWLS contains five items, such as “I am satisfied with my life”. Response options range from 1 = *strongly disagree* to 7 = *strongly agree*. The SWLS is widely used in wellbeing research, and has well-established psychometric properties^80^. Cronbach’s alpha in the current sample was 0.91.

*Personality* was measured by the NEO-PI-R^45,81^. The NEO-PI-R contains 240 items tapping the five general factors of personality, namely neuroticism, extraversion, openness to experience, agreeableness and conscientiousness. Within each of these factors, or domains, the NEO-PI-R measures six facets, or sub-factors (see results section for overview of all 30 facets). Each of these facets is measured by eight items. Response options range from 1 = *strongly disagree* to 5 = *strongly agree*. The NEO-PI-R is a well-established instrument, with sound psychometric properties^41^. In the current sample alphas for the five factors were 0.92 (neuroticism), 0.87 (extraversion), 0.88 (openness), 0.84 (agreeableness) and 0.87 (conscientiousness). Alphas for the facets ranged from 0.47 (C5 self-discipline) to 0.85 (N1 anxiety), with a mean of 0.67.

### Analyses

Correlations were used to examine the bivariate associations between life satisfaction and personality traits and their facets. Next, we used regression analyses to (a) examine the unique contributions from the five broad personality traits, and to (b) identify the facets that are important for the association between personality and life satisfaction. Due to the non-independence of observations within twin pairs we used Generalized Estimating Equations (GEE) to account for the paired structure to obtain correct standard errors and significance levels. Further, to adjust for multiple testing we performed subsequent analyses with Bonferroni correction and the False Discovery Rate (FDR) approach^63^.

Based on the regression analyses we conducted two sets of multivariate biometric analyses to estimate the genetic and environmental contributions to the associations between personality and life satisfaction. The first set examined the relation between the major big five factors and life satisfaction. The second set of analyses focused on the specific facets that uniquely predicted life satisfaction. In order to focus on facets with substantive effects, we chose to retain only facets yielding regression betas >0.10, and with p < 0.01.

Standard Cholesky models^82,83^ were used to estimate the genetic and environmental contributions to variance and covariance in personality and life satisfaction. All models were run with the OpenMx package in R^84^. The biometric models take advantage of the basic premise that MZ twins share 100% of their genes, whereas DZ twins share on average 50% of their segregating genes. Generally, the models allow for estimating three major sources of variance, including additive genetic factors (A), common environment (C) and non-shared environment (E). In addition, non-additive genetic effects (D) may be tested, but are only indicated if the observed MZ-correlations are more than twice the DZ-correlations. A Cholesky model is a structural equation model comprising the measured variables as observed phenotypes and the A, C and E components as latent factors (for illustration see Fig. 1). Models are constrained so that latent A-factors correlate perfectly among MZ-twins, and at 0.5 among DZ-twins. C-factors are correlated at unity for both zygosity groups, and E-factors are by definition uncorrelated. Different models are compared to determine the presence of the genetic and environmental effects (e.g., the fit of an ACE model is compared to an AE model) or sex-differences. In line with standard practice, we tested different types of sex-limitation models^85^. First, common sex-limitation models allow parameter estimates to vary across sex, involving differences in magnitude for genetic and environmental effects. Second, scalar sex-limitation allows the unstandardized variance-covariance matrices to vary across sex, but standardized parameters (e.g., heritabilities) are constrained to be equal. Finally, the sex-limitation models were compared with models having all parameters constrained to equal across sex. To assess models and identify the best fitting model we used the minus2LogLikelihood difference (Δ − 2LL) test, and the Akaike Information Criterion (AIC)^86^.

### Data availability

The dataset analyzed during the current study may be requested from the Norwegian Twin Registry. Restrictions apply to the availability of these data, which were used under license for the current study, and so are not publicly available. Information about data access is available here: https://www.fhi.no/en/studies/norwegian-twin-registry/
